# Outcomes of Isolated Delayed Coarctation of the Aorta Surgery in Adults: Our 25-Year Experience

**DOI:** 10.3390/jcm14124337

**Published:** 2025-06-18

**Authors:** Elif Coşkun Sungur, Emre Demir Benli, Şeref Alp Küçüker, Ahmet Sarıtaş

**Affiliations:** Department of Cardiovascular Surgery, Ministry of Health Ankara Bilkent City Hospital, Cankaya, Ankara 06800, Turkey; benemredemir@gmail.com (E.D.B.); serefalp@yahoo.com (Ş.A.K.); ahsaritas@hotmail.com (A.S.)

**Keywords:** aortic coarctation, adult, surgical procedures, recoarctation, intracardiac procedures, extracardiac procedures

## Abstract

**Background:** The aim of this study was to evaluate the clinical outcomes of adult patients who underwent repair for delayed isolated coarctation of the aorta (CoA). In addition, we aimed to assess the immediate results of the interventions and long-term follow-up data. **Methods:** A total of 119 adult patients who were operated on for CoA and remained under follow-up during a 25-year study period were retrospectively analyzed. The pre-, intra-, and postoperative data of the patients were recorded. The surgical methods applied preoperatively and/or postoperatively were classified based on the primary issue as interventions involving the aorta and those not involving the aorta. **Results:** Of the patients, 81 were males and 28 were females with a mean age of 30.55 ± 10.84 (range: 18 to 67) years. The mean follow-up was 74.79 ± 61.71 (range: 0 to 271) months. A statistically significant difference was found between the presence of pre- and postoperative hypertension and the incidence of postoperative hypertension in patients under the age of 30 (*p* = 0.021 and *p* = 0.039, respectively). A total of 13 patients underwent surgery for recoarctation. The overall rate of additional cardiac surgery was 11.80%. The presence of preoperative hypertension and valve morphology (normal vs. bicuspid) were found to be statistically significant for the need for surgery before and after CoA repair. **Conclusions:** Patients with repaired CoA should be closely monitored due to the lifelong risk of developing complications. In particular, we recommend annual follow-up for patients with BAV.

## 1. Introduction

Coarctation of the aorta (CoA) is typically located at the level of the ductus arteriosus and rarely occurs ectopically in other parts of the aorta. It accounts for nearly 5 to 8% of all congenital heart defects, with an estimated prevalence of 3/10,000 live births [1]. Associated cardiovascular anomalies are common, with bicuspid aortic valve (BAV) being the most frequent, accounting for up to 80% of cases [1].

Since the first surgical repair of CoA in 1945, various open surgical techniques for adult CoA repair have been described in the literature [2,3]. Since the first introduction of balloon angioplasty in 1982, transcatheter repair has been increasingly utilized as an alternative to surgery in older children and adults [4].

Coarctation of the aorta can be diagnosed across a wide age range and with varying degrees of severity. The most common age for surgical repair is six months; however, delayed diagnosis and treatment may occur in cases where the condition is initially overlooked [5,6,7]. In older age groups where CoA cannot be detected or treated during childhood, patients typically present with upper extremity hypertension and/or reduced exercise tolerance [8]. Long-term follow-up frequently reveals valvulopathy, vasculopathy, ventricular dysfunction, hypertension, cerebrovascular disease, and complications at the surgical site [9,10,11,12]. The natural history of unrepaired CoA is often characterized by death in the fifth decade of life due to hypertensive complications [3].

Improved technological imaging and interventional techniques have provided important information for the diagnosis and treatment of coarctation and long-term data accumulation for patients undergoing coarctation repair in childhood. However, surgical experience and long-term follow-up data for delayed isolated aortic coarctations are still very limited [13].

In the present study, we aimed to evaluate the clinical outcomes of adult patients who underwent surgical and/or endovascular treatment for isolated CoA at a high-volume cardiovascular center. In addition, we aimed to assess the immediate results of the initial interventions and long-term follow-up data in order to investigate additional intra- and/or extracardiac procedures performed over time.

## 2. Materials and Methods

### 2.1. Study Design and Study Population

For this retrospective study, patients who underwent different procedures for coarctation of the aorta for the first time or recoarctation between 1999 and 2016 (20 years) in a hospital that served between 1964 and 2019 and between 2019 and 2023 (5 years) in a tertiary care hospital that started to serve as a continuation of the hospital in February 2019 were analyzed. This single-center, retrospective study was conducted at the Department of Cardiovascular Surgery of Ankara Bilkent City Hospital between August 1999–2023. Initially, consecutive patients aged ≥18 years who previously underwent coarctation repair and were under follow-up were screened. All patients with isolated CoA, defined as the absence of associated congenital heart disease except for BAV, persistent ductus arteriosus, or small atrial or ventricular septal defects, were included in the study [13]. Patients with complex concomitant cardiac anomalies were excluded. A total of 259 adult patients who underwent interventions for CoA operated by the same surgeons between 1999–2016 (20 years) and 2019–2023 (5 years) were reviewed. Patient data were retrieved from the hospital database. Patients who were operated on before 1999 were excluded due to the absence of a computerized database system, and those operated on between 2016 and 2019 were also excluded due to changes in the hospital’s operating system which rendered patient records inaccessible. Finally, a total of 119 adult patients with accessible medical records who were under follow-up and underwent various interventions during the study period due to associated intra- and/or extracardiac pathologies were recruited. The pre-, intra-, and postoperative data were recorded. Patient consent was waived on the grounds that the ‘informed consent form’ was not deemed mandatory by the Ethics Committee where the study was approved. The study protocol was approved by the institutional Ethics Committee (No: E1-23-3877, Date: 16 August 2023). The study was conducted in accordance with the principles of the Declaration of Helsinki.

The first postoperative follow-up was performed on the 10th postoperative day, followed by follow-up visits at 1 month, 3 months and 6 months, and once a year after the first year. Control protocols included blood pressure monitoring, outpatient clinic visits with echocardiography at 6 months, and if clinically necessary, contrast-enhanced CT or MRI was planned.

### 2.2. Preoperative Diagnostics and Indication for Surgery

All patients underwent blood pressure measurement, complete blood tests, chest X-ray, electrocardiography, and transthoracic echocardiography (TTE) following pre- and postoperative clinical examinations. For patients suspected of residual or recoarctation, advanced imaging techniques such as contrast-enhanced cardiac computed tomography (CT), contrast-enhanced cardiac magnetic resonance imaging (MRI), and/or catheterization with gradient measurement were performed.

All patients were evaluated for potential clinical risk factors, including hypertension. Resting systemic hypertension was defined as a systolic blood pressure ≥140 mm Hg or diastolic blood pressure ≥90 mm Hg in the right arm, a peak systolic blood pressure >220 mm Hg during maximal exercise in a standard exercise stress test, or the need for antihypertensive medication [14]. After 2020, arterial hypertension was defined according to the European Society of Cardiology (ESC) guidelines as a 24-h ambulatory blood pressure measurement in the right arm showing an average systolic pressure >130 mm Hg and/or diastolic pressure >80 mm Hg [15]. Patients using antihypertensive drugs were considered hypertensive. Residual or recoarctation was defined as a peak gradient ≥25 mm Hg across the repair site on TTE [16]. Additionally, a resting blood pressure gradient of ≥20 mm Hg between the right arm and the leg, a ratio of the transverse aortic arch to the diaphragmatic aorta of <0.7 on contrast imaging, or a repair site to diaphragmatic aorta ratio of <0.7 (repair site diameter ≤70% of diaphragmatic aorta diameter) were also considered indicative of residual or recoarctation [1,16,17].

For the indications of surgical intervention, the updated international guidelines revised between 1999 and 2023 were used as the basis including the 2003 ESC Guidelines for the Management of Adults with Congenital Heart Disease [18], 2008 American College of Cardiology (ACC) and American Heart Association (AHA) Guidelines for the Management of Adults with Congenital Heart Disease [19], and 2010 ESC Guidelines for the Treatment of Adults with Congenital Heart Disease [1]. According to the 2020 ESC Guidelines for the Treatment of Adults with Congenital Heart Disease, the current definition recommends endovascular treatment (stenting) as the preferred first-line procedure, when technically feasible, in hypertensive patients with increased non-invasive blood pressure between the upper and lower extremities and invasively confirmed pressure gradient (>20 mm Hg peak-to-peak) [15].

### 2.3. Study Definitions

Our study focused on major postoperative issues commonly reported in the literature following CoA surgery, including ascending aortic intervention, aortic valve dysfunction requiring intervention, reintervention for recoarctation (arch reintervention), and residual/recurrent systemic hypertension. The surgical methods applied preoperatively and/or postoperatively were classified based on the primary issue as interventions involving the aorta and those not involving the aorta.

Interventions targeting coarctation were defined as surgery for coarctation (SC) and endovascular intervention for coarctation (EIC). Surgical procedures applied to patients for coarctation were categorized as resection and tubular graft interposition (RIPG), patch aortoplasty (PA), shunt using a tubular graft between the left subclavian artery and the descending aorta (SH), and subclavian flap aortoplasty (SFA). Operations before and after coarctation surgery were also classified under the heading “Other” as surgeries involving aortic intervention [aortic root and/or ascending aortic intervention surgery (AIS)] and surgeries without aortic intervention (SAI), which included valve surgeries and/or non-complex congenital operations. Patients who did not undergo any surgery were designated as no intervention/surgery (NO).

The STROBE statement for this observational study is attached (Supplementary Materials). 

### 2.4. Statistical Analysis

Statistical analysis was performed using the SPSS version 30.0 software (IBM Corp., Armonk, NY, USA). Continuous data were presented in mean ± standard deviation (SD) or median (min–max), while categorical data were presented in number and frequency. The categorical data obtained before and after the operation were analyzed using the McNemar test. The Pearson chi-square test, Fisher exact test, and Fisher–Freeman–Halton test were used to analyze the frequency distributions of categorical data in terms of independent groups. A *p* value of <0.05 was considered statistically significant.

## 3. Results

Of the patients, 81 (68.1%) were males and 28 (31.9%) were females with a mean age of 30.55 ± 10.84 (range: 18 to 67) years. The mean follow-up was 74.79 ± 61.71 (range: 0 to 271) months. The most common associated anomaly was BAV in 42 (35.3%) patients.

**Figure 1:**
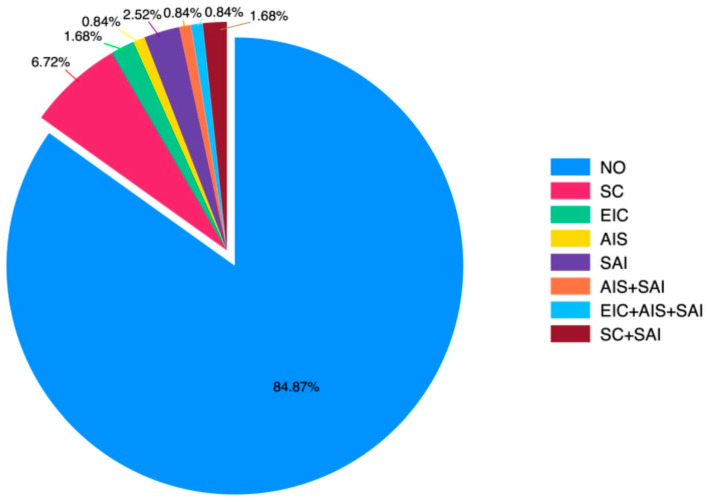
Surgeries prior to CoA surgery. CoA: coarctation of the aorta, AIS: aortic intervention surgery, SAI: surgery without aortic intervention, EIC: endovascular intervention for coarctation, SC: surgery for coarctation. No intervention/surgery (NO).

Preoperative hypertension, aortic valve disorders (stenosis and/or regurgitation), and previous cardiac interventions prior to coarctation surgery with operative details were evaluated. The statistical distribution graph of patients’ history of surgical operations before CoA surgery is presented in Figure 1.

In the preoperative period, hypertension, aortic valve disorders (stenosis and/or regurgitation), and whether any cardiac intervention was performed before coarctation surgery were assessed (Table 1). 

Diagnoses at the time of admission, types of surgeries performed, residual/re-recoarctation, mortality, and morbidity outcomes are summarized in Table 2.

**Figure 2:**
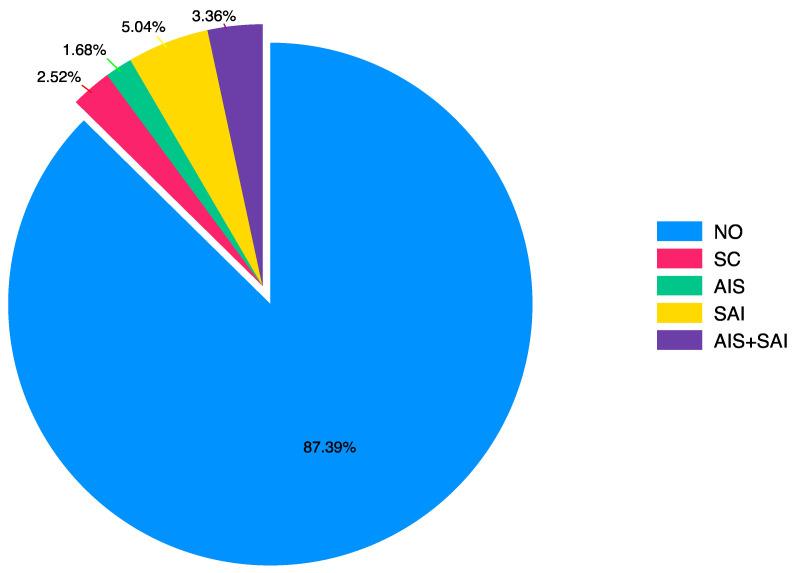
Surgeries after CoA surgery. CoA: coarctation of the aorta, NO: no aortic intervention, AIS: aortic intervention surgery, SAI: surgery without aortic intervention, EIC: endovascular intervention for coarctation, SC: surgery for coarctation.

In the postoperative period, hypertension, aortic valve disorders (stenosis and/or regurgitation), and whether any cardiac intervention was performed after coarctation surgery were assessed (Table 3). The statistical distribution graph of surgical operations after CoA surgery is shown in Figure 2.

A statistically significant difference was found between the presence of hypertension before and after surgery (*p* = 0.021). Hypertension was observed in 78 (65.55%) patients before surgery, whereas the number of patients with hypertension after surgery was 68 (57.14%) (Table 4).

The incidence of hypertension before and after surgery varied according to age groups. Among patients older than 30 years, no statistically significant change was observed in the incidence of hypertension before and after surgery (*p* = 0.625). However, in patients younger than 30 years, this difference was statistically significant (*p* = 0.039). Among the patients aged below 30 years, hypertension was observed in 40 (58.82%) patients before surgery and in 32 (47.06%) patients after surgery (Table 5).

Throughout the study period, 56.6% of patients undergoing coarctation surgery received RIPG, 37.7% received PA, 4.7% received SH, and 0.9% received SFA. Thirteen of these patients underwent recoarctation surgery, with three (23.1%) patients receiving PA and the others (76.9%) receiving RIPG (Table 6).

Considering the factors potentially related to pre-CoA surgical reintervention, the presence of preoperative hypertension and valve morphology (normal vs. bicuspid) were found to be statistically significant (*p* < 0.05) (Table 7). Considering the factors potentially related to post-CoA surgical reintervention, sex, presence of pre- and postoperative hypertension, and valve morphology (normal vs. bicuspid) were found to be statistically significant factors (*p* < 0.05) (Table 8).

The distribution of preferred surgical procedures between 1999 and 2023 is shown in Table 9. Accordingly, the rate of RIPG application increased, while the rate of PA application decreased over the years.

## 4. Discussion

In certain countries, including Türkiye, the surgical treatment of CoA may be delayed until adulthood and, unfortunately, may only be performed after irreversible changes have already occurred. To the best of our knowledge, the present study represents the largest adult cohort in the literature to report long-term outcomes of isolated CoA surgical repair, both in Türkiye and globally.

To date, several authors have reported their experiences with the surgical treatment of CoA in adults. Recent studies have shown low mortality and morbidity rates associated with the surgical procedure choice for CoA. A recent study reported a 30-day mortality rate of 0.54% [20]. However, it has been well established that repairing coarctation does not warrant a cure, and late cardiac morbidity may still develop after the repair [11,21,22,23,24]. Heart failure with preserved ejection fraction in adult patients with corrected CoA is explained by the persistence of frequent endothelial dysfunction of the aorta despite repair and a chronic increased afterload leading to first left and then right ventricular dysfunction [25,26]. One cohort study demonstrated that only 66% of patients survived to the age of 70 [27]. Another study on post-repair survival suggested that more than 25% of patients may die within 30 years following the repair [28]. Moreover, nearly half of the patients require further invasive cardiovascular interventions by the age of 50 [27,29,30].

The presence of hypertension in the postoperative period constitutes a significant risk factor for mortality, as deaths are often associated with conditions such as heart failure and aortic aneurysm rupture [31]. In the literature, postoperative systemic hypertension is reported in 60 to 75% of patients after coarctation repair [27,28]. In our cohort, the frequency of hypertension following surgery was found to be 57.14%, which is consistent with other studies indicating persistent late-onset hypertension in patients with repaired CoA [11,13,32,33]. Well-recognized potential mechanisms for the development of late hypertension reported after coarctation repair include patient-related (older age, longer follow-up), surgical (coarctation repair at an advanced age), mechanical (recoarctation) and metabolic (increased sympathetic activity, systemic vasculopathy) causes [9,11,28,34,35,36,37].

In the current study, the mean age of the patients was 30.55 ± 10.84 (range: 18 to 67) years. Primary repairs in these patients were performed at a significantly older average age than reported in previously published data [13,21,23,27,28,38]. In our cohort, regardless of age group, there was a statistically significant difference between the pre- and postoperative incidence of hypertension. Although the surgical procedure performed reduced the overall frequency of postoperative hypertension, postoperative hypertension was age-dependent at the time of surgery. In our study, among patients who underwent surgery before and after the age of 30, there was a statistically significant difference in favor of those who were operated on before age 30, both in terms of pre- and postoperative hypertension. This finding supports the view that an older age at the time of initial repair is associated with irreversible vascular dysfunction [33,35,39].

Considering the factors that may be associated with undergoing surgery before CoA repair, the presence of preoperative hypertension was found to be statistically significant in our study. Among patients who underwent open-heart surgery prior to CoA repair, preoperative hypertension was identified in 94.40% of cases. Considering the factors that may be associated with undergoing surgery after CoA repair, both pre- and postoperative hypertension were found to be statistically significant. Among patients who underwent open-heart surgery following CoA repair, 100% had preoperative hypertension and 85% had postoperative hypertension. In one of the largest series reporting experiences with CoA, residual/recoarctation was identified in 60% of patients with postoperative hypertension [27]. In addition, nearly 60% of the patients developed systemic hypertension which was often unrelated to the repair status. In our results, the frequency of recoarctation in patients with hypertension after coarctation repair was found to be 13.20%. As shown in this study, recoarctation accounts for only a small proportion of postoperative hypertension cases. Additionally, among patients with postoperative hypertension, 17.60% of the patients underwent a subsequent operation after CoA repair. Based on our findings, we believe that hypertension observed particularly in the postoperative period should be evaluated and managed independently from the corrected coarctation pathology.

Following CoA surgery, recoarctation rates of up to 60% have been reported in the literature [27]. Yousif et al. [40] shared their experience treating 38 adult patients with interposition grafting, nearly half of whom had previously repaired CoA. Over nearly 10 years of follow-up, they reported no cases of recoarctation. In their study including 60 adolescent and adult patients, Yin et al. [41] predominantly used a non-anatomical bypass technique and performed a concomitant cardiac procedure in 28% of cases. Among these patients, 18% underwent RIPG, and no recoarctation was reported over a follow-up period of 45.3 months. Another report by Charlton-Ouw et al. [3] including 29 adult patients showed that, in cases where CoA was associated with thoracic aortic aneurysms, the interposition graft technique was preferred in 62% of patients. During an average follow-up of 81 months, no recoarctation was observed. Abjigitova et al. [13] performed surgical reconstruction mainly using the end-to-end anastomosis technique in 90 patients, 17% of whom underwent RIPG. During an average follow-up of 26.8 years, they reported a 4% recoarctation rate. In a study by Lee et al. [27] involving 834 patients, more than half of whom were treated using the end-to-end anastomosis technique, 7% underwent RIPG. Over a mean follow-up period of 27 years, they reported a 60% rate of residual/recoarctation.

In our study, we observed that the choice of surgical procedure has evolved over time due to complications such as recoarctation and aneurysms. While patch aortoplasty was preferred in the earlier years, tubular graft interposition has become the favored technique in recent years. Among adults, our preferred open surgical technique is RIPG. Although our study represents one of the largest RIPG series in adult patients, there is currently insufficient data to appropriately compare different repair techniques. While the exact causes of recoarctation remain unclear, studies comparing these surgical techniques are highly heterogeneous, making it difficult to claim the superiority of one approach over another [31]. In recent years, the importance of tailoring the surgical technique to the individual patient has gained increasing recognition [42].

The incidence of BAV (bicuspid aortic valve) in our cohort was 35.3% (119/42), which is lower than the frequency reported in similar studies [3,13,27,43]. The association between CoA and BAV is clinically significant, as it can lead to progressive aortic root dilation, necessitating aortic valve surgery and, to a lesser extent, ascending aortic surgery [27,31]. In our study, 14 patients underwent such postoperative surgeries, and BAV was identified in 11 of them (78.6%). Considering the frequency distribution between BAV and ascending aortic aneurysms, valve morphology showed a statistically significant impact on the likelihood of requiring reoperation after the initial surgery. This difference seems to originate from patients diagnosed with BAV. As shown in Table 8, 78.6% of the patients who underwent postoperative reoperation had BAV.

Considering the factors potentially associated with undergoing surgery prior to CoA repair, aortic valve morphology (normal vs. bicuspid) was found to be statistically significant in the current study. In patients who required reoperation following coarctation repair, the incidence of preoperative aortic regurgitation was identified as 23.8%. Among patients diagnosed with postoperative recoarctation, the frequency of preoperative aortic regurgitation was observed in approximately 46.6% of cases. Additionally, BAV was detected in 52.4% of patients with preoperative aortic regurgitation. No significant relationship was observed between other valve dysfunctions and the presence of residual/recoarctation or the need for reoperation. Based on our findings, we believe that aortic valve morphology plays a key role in CoA repair.

When congenital heart disease is accompanied by a BAV, it not only increases the frequency of aortopathy involving the ascending aorta, but is also reported to contribute to the need for repeat cardiac surgery [23]. In the study by Abjigitova et al. [13], which included 90 patients, the need for additional cardiac surgery following coarctation repair was reported at 30%. In this patient population, where the incidence of BAV was 43%, aortic valve replacement was performed in 41.9% of the reintervention cases. In the study by Lee et al. [27], which included 834 patients with a BAV incidence of 58%, 30% of patients (246/834) required ≥1 arch reintervention, 13% (111/834) required aortic valve intervention, and 5% (43/834) required ascending aortic intervention. Bicuspid aortic valve was identified as an independent risk factor for ascending aortic intervention. In the study by Choudhary et al. [43], no correlation was found between the presence of BAV and ascending aortic aneurysms or the need for repeat cardiac surgical procedures, which was attributed to the limited sample size. In our series, where the BAV incidence was low, the requirement for additional cardiac surgery, even when recoarctation cases were included, was found to be 11.8%, which is considerably lower than the rates reported in similar studies.

Current studies report reoperation rates ranging from 3 to 40% with either surgical or catheter-based techniques [21,28,44]. Among the patients who presented with recoarctation, only three (2.5%) previously underwent endovascular intervention, and in our series, none of the patients received an endovascular procedure. Endovascular repair in the treatment of CoA is reported to be safe and effective as it is associated with a high procedural success rate, an acceptable incidence of complications and recoarctation [45,46]. More than half of the patients who underwent coarctation surgery during the study period underwent prior RIPG procedures. Although patch aortoplasty is not usually recommended due to the high incidence of aortic aneurysms [47], none of the patients in our study who underwent patch aortoplasty (*n* = 40) aneurysms. Upon their initial admission to our center, 106 patients (89.1%) were diagnosed with coarctation for the first time, while 13 patients (10.9%) were diagnosed with recoarctation. Following surgical treatment, three of our patients (2.5%) were diagnosed with recoarctation. We believe that the low rates of recoarctation and the need for additional cardiac surgery in our series are due to the limited patient group with isolated CoA and the low incidence of BAV, without complex cardiac pathologies.

The role of early coronary artery disease (CAD) in repaired CoA still remains controversial in the literature. In a study including patients with CoA and the general population, it was reported that patients with CoA required coronary revascularization 15 years earlier than those without CoA [31]. However, two studies that specifically investigated the independent risk of early CAD in CoA patients found no such association [48,49]. Similarly, our study did not identify any such relationship.

### Limitations

Since our cohort spans a 25-year period, our results reflect both historical and contemporary practices. The current study is a retrospective, single-center analysis of a limited and selected group of consecutive patients. Nevertheless, it represents one of the largest populations analyzed for primary adult CoA and contributes to the understanding of long-term outcomes following surgical repair. As our study used single-center data, the findings may not accurately represent patient outcomes in other hospitals or different healthcare settings.

The main limitation of this study is that 54% of our surgically treated patients were excluded due to the unavailability of hospital records. Only data from a limited number of patients who could be contacted and who underwent intervention during adulthood were included. Furthermore, patients with complex cardiac pathologies beyond isolated coarctation were excluded from the study. Therefore, our findings may not fully reflect the total morbidity burden of all survivors following coarctation surgery. There is a possibility of selection bias as we were only able to include patients who could be contacted and had complete records for interventions performed in adulthood. Additionally, as this is a retrospective evaluation of a 25-year experience by the same surgical team, we were unable to assess the types of grafts, patches, or suture materials used during the operations. A more in-depth analysis of how this limiting situation might contribute to the assessed results could not be carried out. Finally, the number of patients who underwent endovascular repair in our study was insufficient to reliably and effectively evaluate patient outcomes.

## 5. Conclusions

In conclusion, patients with CoA remain at high cardiovascular risk throughout their lives due to the adverse cardiovascular effects of abnormal hemodynamics and potential systemic vascular dysfunction. We believe that all clinicians should recognize the importance of lifelong follow-up in patients with repaired coarctation. Furthermore, considering the possibility that these patients may require aortic valve and ascending aorta interventions in the future, particularly those with BAV, we consider regular close monitoring on an annual basis to be essential. Considering that the findings of our study included descriptive characteristics, we think that our results should be considered hypothesis-forming. It is evident that phenotype-based, randomized-controlled studies involving heterogeneous patient populations are warranted to improve survival and to better identify risk factors for potential future cardiovascular diseases.

## Figures and Tables

**Table 1:** Preoperative data of the patients.

%	*n*		
34.50%	41	No	Preoperative hypertension
65.50%	78	Yes	
90.80%	108	None	Preoperative aortic stenosis
5.00%	6	Mild	
4.20%	5	Moderate	
0.00%	0	Severe	
70.60%	84	None	Preoperative aortic regurgitation
13.40%	16	Mild	
14.30%	17	Moderate	
1.70%	2	Severe	
84.90%	101	No	Surgical intervention before CoA surgery
15.10%	18	Yes	
10.90%	13	CoA targeted	
6.70%	8	SC	
1.70%	2	EIC	
2.5%	3	Simultaneous with CoA	
0.80%	1	EIC + AIS + SAI	
1.70%	2	SC + SAI	
4.20%	5	Other	
0.80%	1	AIS	
2.50%	3	SAI	
0.80%	1	AIS + SAI	

CoA: coarctation of the aorta, AIS: aortic intervention surgery, SAI: surgery without aortic intervention, EIC: endovascular intervention for coarctation, SC: surgery for coarctation.

**Table 2:** Operative and postoperative data of the patients.

%	*n*		
89.10%	106	CoA	Diagnosis
10.90%	13 (12 + 1 ^a^)	Re-CoA	
58.80%	70	RIPG	Surgery type
36.10%	43	PA	
4.20%	5	SH	
0.80%	1	SFA	
87.40%	104	No	Postoperative recoarctation
12.60%	15 (12 + 1 ^a^ + 2 ^b^)	Yes	
20.00%	3 (1 ^a^ + 2 ^b^)	Our center	Undergoing recoarctation surgery
80.00%	12	External center	
98.30%	117	No	Mortality
1.70%	2	Yes	
89.90%	107	No	Morbidity
10.10%	12	Yes	

CoA: coarctation of the aorta, RIPG, resection and interposition of a tubular graft, PA: patch aortoplasty, SH: shunt with a tubular graft between the left subclavian artery and the descending aorta, SFA: subclavian flap aortoplasty. 1 ^a^: One patient developed recoarctation after the initial surgery performed at our clinic. At the time of the second admission, there was a known history of recoarctation. 2 ^b^: Two patients developed recoarctation after their initial surgeries performed at our clinic. At the time of admission, there was no known history of recoarctation.

**Table 3:** Postoperative data of the patients.

%	*n*		Postoperative Signs and Symptoms
42.90%	51	No	Postoperative hypertension
57.10%	68	Yes	
92.40%	110	None	Postoperative aortic stenosis
3.40%	4	Mild	
2.50%	3	Moderate	
1.70%	2	Severe	
77.30%	92	None	Postoperative aortic regurgitation
10.10%	12	Mild	
6.70%	8	Moderate	
5.90%	7	Severe	
88.20%	105	No	Surgical intervention after CoA surgery
11.80%	14	Yes	
2.52%	3	SC	
10.10%	12	Other	
1.70%	2	AIS	
5.00%	6	SAI	
3.40%	4	AIS + SAI	

CoA: coarctation of the aorta, AIS: aortic intervention surgery, SAI: surgery without aortic intervention, SC: surgery for coarctation.

**Table 4:** Pre- and postoperative hypertension status of the patients.

	Postoperative HT						
	Total		No		Yes		
*p*	%	*n*	%	*n*	%	*n*	Preoperative HT
	65.55%	78	16.67%	13	83.33%	65	Yes
**0.021**	34.45%	41	92.68%	38	7.32%	3	No
	100%	119	42.86%	51	57.14%	68	Total

HT: hypertension.

**Table 5:** Pre- and postoperative hypertension status according to age groups.

	Postoperative HT							
	Total		No		Yes			
*p*	%	*n*	%	*n*	%	*n*	Preoperative HT	Age Group
**0.04**	**58.82**%	**40**	25.00%	10	75.00%	30	Yes	18–30 years
	41.18%	28	92.86%	26	7.14%	2	No	
	100%	68	52.94%	36	**47.06**%	**32**	Total	
0.63	**74.51**%	**38**	7.89%	3	92.11%	35	Yes	>30 years
	25.49%	13	92.31%	12	7.69%	1	No	
	100%	51	29.41%	15	**70.59**%	**36**	Total	

HT: hypertension.

**Table 6:** Diagnoses and surgery types.

		Diagnosis				
Total		Re-CoA		CoA		
%	*n*	%	*n*	%	*n*	Surgery Type
58.80%	70	76.90%	10	56.60%	60	RIPG
36.10%	43	23.10%	3	37.70%	40	PA
4.20%	5	0.00%	0	4.70%	5	SH
0.80%	1	0.00%	0	0.90%	1	SFA

CoA: coarctation of the aorta, RIPG, resection and interposition of a tubular graft, PA: patch aortoplasty, SH: shunt with a tubular graft between the left subclavian artery and the descending aorta, SFA: subclavian flap aortoplasty.

**Table 7:** Surgical interventions prior to CoA surgery.

	Operation Prior to CoA Surgery					
*p*	No		Yes			
	%	*n*	%	*n*		
0.34	66.30%	67	77.80%	14	Male	Sex
	33.70%	34	22.20%	4	Female	
0.51	58.40%	59	50.00%	9	18–30 years	Age group
	41.60%	42	50.00%	9	>30 years	
**0.01**	39.60%	40	5.60%	1	No	Preoperative HT
	60.40%	61	94.40%	17	Yes	
**0.01**	66.30%	67 b	44.40%	8 a	Normal	Valve morphology
	33.70%	34 b	44.40%	8 a	BAV	
	0.00%	0 a	11.10%	2 a	AVR	

CoA: coarctation of the aorta, CoA: coarctation of the aorta, BAV: bicuspid aortic valve, AVR: aortic valve replacement, HT: hypertension. a, b: Different subscript letters in the same row indicate a statistically significant difference between column percentages within the subcategories of each variable (*p* < 0.05).

**Table 8:** Surgical interventions after CoA surgery.

	Operation After CoA Surgery					
*p*	No		Yes			
	%	*n*	%	*n*		
**0.005**	63.80%	67	100.00%	14	Male	Sex
	36.20%	38	0.00%	0	Female	
0.998	57.10%	60	57.10%	8	18–30 years	Age group
	42.90%	45	42.90%	6	>30 years	
**0.002**	39.00%	41	0.00%	0	No	Preoperative HT
	61.00%	64	100.00%	14	Yes	
**0.041**	46.70%	49	14.30%	2	No	Postoperative HT
	53.30%	56	85.70%	12	Yes	
**0.014**	68.60%	72 b	21.40%	3 a	Normal	Valve morphology
	29.50%	31 b	78.60%	11 a	BAV	
	1.90%	2 a	0.00%	0 a	AVR	

CoA: coarctation of the aorta, CoA: coarctation of the aorta, BAV: bicuspid aortic valve, AVR: aortic valve replacement, HT: hypertension. a, b: Different subscript letters in the same row indicate a statistically significant difference between column percentages within the subcategories of each variable (*p* < 0.05).

**Table 9:** Surgery types according to years.

	Year of Surgery						
		2019–2023		2009–2018		1999–2008	Surgery Type
*p*	%	*n*	%	*n*	%	*n*	
	**82.76**%	24 b	**66.67**%	24 b	40.74%	22 a	RIPG
	13.79%	4 b	27.78%	10 b	**53.70**%	29 a	PA
	0.00%	0 a	5.56%	2 a	5.56%	3 a	SH
	3.45%	1 a	0.00%	0 a	0.00%	0 a	SFA
**<0.001**	100.00%	29	100.00%	36	100.00%	54	Total (*n*)

CoA: coarctation of the aorta, RIPG, resection and interposition of a tubular graft, PA: patch aortoplasty, SH: shunt with a tubular graft between the left subclavian artery and the descending aorta, SFA: subclavian flap aortoplasty. a, b: Different subscript letters in the same row indicate a statistically significant difference between column percentages within the subcategories of each variable (*p* < 0.05).

## Data Availability

The data that support the findings of this study are available on request from the corresponding author. The data are not publicly available due to privacy or ethical restrictions.

## Supplementary Materials

The following supporting information can be downloaded at: https://www.mdpi.com/article/10.3390/jcm14124337/s1
